# The effects of antenatal dietary and lifestyle advice for women who are overweight or obese on neonatal health outcomes: the LIMIT randomised trial

**DOI:** 10.1186/s12916-014-0163-9

**Published:** 2014-10-13

**Authors:** Jodie M Dodd, Andrew J McPhee, Deborah Turnbull, Lisa N Yelland, Andrea R Deussen, Rosalie M Grivell, Caroline A Crowther, Gary Wittert, Julie A Owens, Jeffrey S Robinson, For the LIMIT Randomised Trial Group

**Affiliations:** The University of Adelaide, School of Paediatrics and Reproductive Health, Robinson Research Institute, Adelaide, South Australia Australia; The Women’s and Children’s Hospital, Women’s and Babies Division, Department of Perinatal Medicine, North Adelaide, South Australia Australia; The Women’s and Children’s Hospital, Women’s and Babies Division, Department of Neonatal Medicine, Adelaide, South Australia Australia; The University of Adelaide, School of Psychology, Adelaide, South Australia Australia; Women’s and Children’s Health Research Institute, North Adelaide, South Australia Australia; The University of Adelaide, School of Population Health, Adelaide, South Australia Australia; The University of Adelaide, School of Medicine, Adelaide, South Australia Australia; Liggins Institute, The University of Auckland, Auckland, New Zealand

**Keywords:** Pregnancy, Overweight and obesity, Neonatal health, Randomised trial, Dietary and lifestyle intervention

## Abstract

**Background:**

Overweight and obesity during pregnancy represents a considerable health burden. While research has focused on interventions to limit gestational weight gain, there is little information describing their impact on neonatal health. Our aim was to investigate the effect on a range of pre-specified secondary neonatal outcomes of providing antenatal dietary and lifestyle advice to women who are overweight or obese.

**Methods:**

We report a range of pre-specified secondary neonatal outcomes from a large randomised trial in which antenatal dietary and lifestyle advice was provided to women who were overweight or obese. Pregnant women were eligible for participation with a body mass index of 25 kg/m^2^ or over, and singleton gestation between 10^+0^ and 20^+0^ weeks. Outcome measures included gestational age at birth; Apgar score below 7 at 5 minutes of age; need for resuscitation at birth; birth weight above 4.5 kg or below 2.5 kg; birth weight, length and head circumference (and Z-scores); admission to the nursery; respiratory distress syndrome; and postnatal length of stay. Data relating to the primary outcome (large for gestational age infants defined as birth weight above the 90th centile) and birth weight above 4 kg have been reported previously. Analyses used intention-to-treat principles.

**Results:**

In total, 2,142 infants were included in the analyses. Infants born to women following lifestyle advice were significantly less likely to have birth weight above 4.5 kg (2.15% versus 3.69%; adjusted risk ratio (aRR) = 0.59; 95% confidence interval (CI) 0.36 to 0.98; *P* = 0.04), or respiratory distress syndrome (1.22% versus 2.57%; aRR = 0.47; 95% CI 0.24 to 0.90; *P* = 0.02), particularly moderate or severe disease, and had a shorter length of postnatal hospital stay (3.94 ± 7.26 days versus 4.41 ± 9.87 days; adjusted ratio of means 0.89; 95% CI 0.82 to 0.97; *P* = 0.006) compared with infants born to women who received Standard Care.

**Conclusions:**

For women who are overweight or obese, antenatal dietary and lifestyle advice has health benefits for infants, without an increase in the risk of harm. Continued follow-up into childhood will be important to assess the longer-term effects of a reduction in high infant birth weight on risk of child obesity.

Please see related articles: http://www.biomedcentral.com/1741-7015/12/161 and http://www.biomedcentral.com/1741-7015/12/201.

**Clinical trial registration:**

Australian and New Zealand Clinical Trials Registry (ACTRN12607000161426)

## Background

Globally, it is estimated that 170 million children under the age of 18 years [1], are overweight or obese. Obesity is occurring at an increasingly early age, affecting more than 43 million children aged 0 to 5 years world-wide [2], and 21% of Australian children 2 to 3 years of age [3]. The World Health Organization has described childhood obesity as “one of the most serious public health challenges of the 21st century”, [4] with obese children exposed to its consequences, including disease progression and disability, earlier and for longer duration.

The economic costs of childhood obesity are profound [5]. Australian data indicate that children who are overweight or obese at 5 years of age have medical costs within the first 5 years of school that are $9.8 million higher than those of children of normal body mass index (BMI) [6]. Data from the USA indicate that childhood overweight and obesity are associated with an additional cost of $14.1 billion annually, reflecting prescription drugs and emergency and outpatient attendances [7], with a further $238 million annually reflecting inpatient admissions [8]. The direct medical costs, in both childhood and adulthood, directly attributable to high childhood BMI have been conservatively estimated to be $6.24 billion, with over 2 million quality adjusted life years lost [5].

The intra-uterine environment is recognised as playing a key role in the development of later health and disease [9], representing a crucial period in the subsequent programming of obesity. Both high maternal BMI and excessive gestational weight gain have been consistently associated with adverse pregnancy outcomes [10-13], and are significant predictors of increased adiposity and future child/adult obesity [14-17], with some studies also finding consequent associations with cardiometabolic risk factors, including higher blood pressure [18,19]. The antenatal period therefore represents a unique window in which intervention designed to alter maternal diet and weight gain may significantly influence infant adiposity, and modify future risk of both child and adulthood obesity.

Although there is considerable research focused on the effects of dietary and lifestyle interventions to limit gestational weight gain by pregnant women who are overweight or obese, their effect on neonatal outcomes has been poorly reported in the literature to date [20-22]. In the few studies specifically involving women who are overweight or obese where birth outcomes have been reported, the predominant focus has been on infant birth weight, with no reporting of other relevant clinical infant outcomes [20-22]. We report the findings of the LIMIT randomised trial, evaluating the provision of antenatal dietary and lifestyle advice to women who were overweight or obese on a range of pre-specified secondary neonatal health outcomes.

## Methods

### Ethics

Ethics approval was granted by the Women’s and Children’s Local Health Network Human Research and Ethics Committee at the Women’s and Children’s Hospital, the Central Northern Adelaide Health Service Ethics of Human Research Committee (Lyell McEwin Hospital) and the Flinders Clinical Research Ethics Committee (Flinders Medical Centre). Approval to conduct the trial was provided by the Human Research and Ethics Committee at each participating centre, and all participants provided written informed consent.

### Study design

We conducted a multicentre randomised trial across the three major metropolitan maternity hospitals within Adelaide, South Australia. The methods [23] and primary findings [24] of the LIMIT randomised trial have been reported previously, and the trial has been registered on the Australian and New Zealand Clinical Trials Registry (ACTRN12607000161426). Additional clinical neonatal outcomes were added to the final working protocol, reflecting piloting of data collection processes. These amendments were pre-specified in the final working protocol, early in the conduct of the trial, and prior to any analyses being undertaken.

### Inclusion and exclusion criteria

Women with a BMI of 25 kg/m^2^ or greater and singleton pregnancy between 10^+0^ and 20^+0^ weeks gestation were eligible to participate in the trial. Women with a multiple pregnancy, or type 1 or 2 diabetes diagnosed prior to pregnancy were ineligible.

### Trial entry

All women had their height and weight measured and their BMI calculated at their first antenatal appointment, and eligible women were counselled about participation.

### Randomisation, masking and group allocation

Randomisation occurred by telephoning the central randomisation service, using a computer-generated schedule, with balanced variable blocks, and stratification for parity (0 versus ≥1), BMI at antenatal booking (25 to 29.9 kg/m^2^ versus ≥30 kg/m^2^), and collaborating centre. Women were randomised and allocated to either ‘Lifestyle Advice’ or ‘Standard Care’.

### Intervention

#### Lifestyle advice group

Women randomised to receive Lifestyle Advice participated in a comprehensive dietary and lifestyle intervention over the course of their pregnancy, which included a combination of dietary, exercise and behavioural strategies, delivered by a research dietician and trained research assistants [23]. Women were provided with dietary advice consistent with current Australian standards [25]; to maintain a balance of carbohydrates, fat and protein, to reduce intake of foods high in refined carbohydrates and saturated fats, while increasing intake of fibre, and to promote consumption of two servings of fruit, five servings of vegetables, and three servings of dairy each day [25]. Physical activity advice primarily encouraged women to increase their amount of walking and incidental activity [26]. The content and structure of the intervention sessions has been described in detail previously [24].

#### Standard care group

Women randomised to receive Standard Care continued their pregnancy care according to local hospital guidelines, which did not include routine provision of advice related to diet, exercise or gestational weight gain.

### Study outcomes

In clinical practice, there is considerable variation in definitions of ‘large for gestational age’, including birth weight at or above the 90th centile for gestational age and infant sex, birth weight above 4 kg, and birth weight above 4.5 kg, which are often used interchangeably. These have been recognised as associated with early childhood obesity [18,27], and were chosen as outcome measures in the LIMIT randomised trial. The incidence of infants born large for gestational age (birth weight ≥90th centile for gestational age and infant sex; primary outcome), and with birth weight above 4 kg have been reported previously [24]. Pre-specified secondary neonatal outcomes included gestational age at birth; Apgar score of 7 or above at 5 minutes of age; need for resuscitation at birth; birth weight above 4.5 kg or below 2.5 kg; birth weight (and Z-scores); birth length (and Z-scores); head circumference (and Z-scores); admission to neonatal intensive care unit; admission to special care baby unit; respiratory distress syndrome [28] (with moderate or severe disease defined as mean airway pressure >10 cm H_2_O and/or inspired oxygen fraction (FiO_2_) >0.80 with ventilation); proven systemic infection requiring treatment; retinopathy of prematurity; necrotising enterocolitis; neonatal encephalopathy [29]; seizures; and postnatal length of stay.

Ponderal Index was calculated using birth weight and length (kg/m^3^). Predicted fat free mass was calculated using the following formula:

$$ 0.507+0.646\times \mathrm{weight}\ \left(\mathrm{kg}\right)\hbox{-} 0.089\times \mathrm{sex}+0.009\times \mathrm{length}\ \left(\mathrm{cm}\right), $$

where 1 = male and 2 = female [30].

### Analysis and reporting of results

Analyses were performed on an intention-to-treat basis, according to the treatment group allocated at randomisation. Multiple imputation was performed separately by treatment group, using chained equations to create 100 complete datasets for analysis. Women who withdrew consent to use their data, or had a miscarriage, termination of pregnancy, or stillbirth, were excluded from the imputation and analysis. Sensitivity analyses were performed using the available data and different imputation models. Binary outcomes were analysed using log binomial regression, with treatment effects expressed as relative risk (RR), or Fisher’s exact test with no imputation for rare outcomes. Continuous outcomes were analysed using linear regression, with treatment effects expressed as differences in means. Count outcomes were analysed using Poisson regression, or using negative binomial regression where over-dispersion was present, with treatment effects expressed as ratios of means.

Both unadjusted and adjusted analyses were performed, with adjustment for the stratification variables. Outcomes derived from birth weight were additionally adjusted for maternal age, socioeconomic status and maternal smoking. Statistical significance was considered at *P* < 0.05 (two-sided) with no adjustment for multiple comparisons. All analyses followed a pre-specified statistical analysis plan and were performed using SAS software (v9.3; SAS Inc., Cary, NC, USA).

### Sample size

Our predetermined sample size of 2,180 women was based on our primary trial outcome, the incidence of large for gestational age infants [24].

## Results

**Figure 1:**
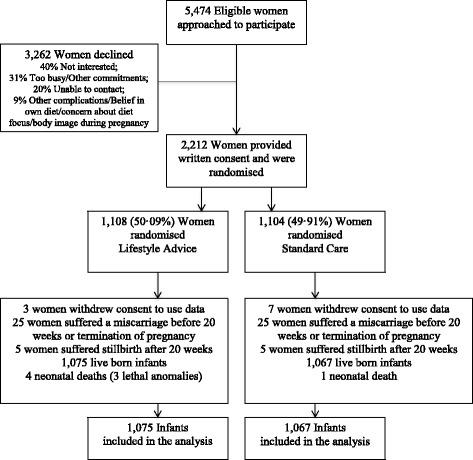
**Flow of participants through the trial.**

Between June 2008 and December 2011, we recruited and randomised 2,212 women, with 1,108 allocated to receive Lifestyle Advice, and 1,104 Standard Care (Figure 1). There was a total of 2,142 live-born infants included in the analyses (1,075 Lifestyle Advice; 1,067 Standard Care). The characteristics of women at the time of randomisation were similar between treatment groups (Table 1).

**Table 1:** **Demographic and clinical characteristics at trial entry (baseline)**

**Characteristic**	**Lifestyle advice (n = 1105** ^**a**^ **)**	**Standard care (n = 1097** ^**a**^ **)**	**Total (n = 2202** ^**a**^ **)**
Maternal age, years^b^	29.3 ± 5.4	29.6 ± 5.6	29.4 ± 5.5
Gestational age at entry, weeks^c^	14.0 (11.9 to 17.0)	14.1 (11.9 to 17.0)	14.1 (11.9 to 17.0)
Body mass index, kg/m^2c^	31.0 (28.1 to 35.9)	31.1 (27.7 to 35.6)	31.1 (27.9 to 35.8)
Body mass index category^d^			
25.0 to 29.9	458 (41.4)	468 (42.7)	926 (42.1)
30.0 to 34.9	326 (29.5)	318 (29.0)	644 (29.2)
35.0 to 39.9	202 (18.3)	183 (16.7)	385 (17.5)
≥40.0	119 (10.8)	128 (11.7)	247 (11.2)
Public patient^c^	1081 (97.8)	1067 (97.3)	2148 (97.5)
Weight, kg^b^	88.6 ± 17.3	88.2 ± 17.6	88.4 ± 17.4
Height, cm^b^	164.9 ± 16.6	164.8 ± 16.5	164.8 ± 16.6
Race^c^			
Caucasian	995 (90.0)	998 (91.0)	1993 (90.5)
Asian	26 (2.4)	34 (3.1)	60 (2.7)
Indian	40 (3.6)	35 (3.2)	75 (3.4)
Other	44 (4.0)	30 (2.7)	74 (3.4)
Smoker^c^	154 (13.9)	126 (11.5)	280 (12.7)
Nulliparous^c^	457 (41.4)	441 (40.2)	898 (40.8)
Previous preterm birth^c^	57 (5.2)	59 (5.4)	116 (5.3)
Previous pre-eclampsia^c^	46 (4.2)	51 (4.6)	97 (4.4)
Previous stillbirth^c^	13 (1.2)	6 (0.5)	19 (0.9)
Previous neonatal death^c^	11 (1.0)	7 (0.6)	18 (0.8)
Previous caesarean section^c^	197 (17.8)	214 (19.5)	411 (18.7)
Family history of diabetes^c^	288 (26.1)	290 (26.4)	578 (26.2)
Family history of hypertension^c^	389 (35.2)	369 (33.6)	758 (34.4)
Family history of heart disease^c^	187 (16.9)	179 (16.3)	366 (16.6)
Index of socio-economic disadvantage^e^			
Unknown	2 (0.2)	1 (0.1)	3 (0.1)
Quintile 1, (most disadvantaged)	340 (30.8)	321 (29.3)	661 (30.0)
Quintile 2	271 (24.5)	264 (24.1)	535 (24.3)
Quintile 3	173 (15.7)	174 (15.9)	347 (15.8)
Quintile 4	150 (13.6)	178 (16.2)	328 (14.9)
Quintile 5, (least disadvantaged)	169 (15.3)	159 (14.5)	328 (14.9)

^a^Includes all women randomised who did not withdraw consent to use their data.

^b^Mean ± standard deviation.

^c^Median (interquartile range).

^d^n (%).

^e^Socioeconomic index as measured by SEIFA (socioeconomic indexes for areas [31]).

There were no statistically significant differences identified between the two treatment groups with regards to gestational age at birth (Lifestyle Advice 39.29 ± 1.74 weeks versus Standard Care 39.23 ± 2.07 weeks; adjusted difference in means 0.07; 95% confidence interval (CI) 0.10 to 0.23; *P* = 0.42) (Table 2). However, infants born to women allocated to Lifestyle Advice were less likely to weigh above 4.5 kg (Lifestyle Advice 2.15% versus Standard Care 3.69%; adjusted risk ratio (aRR) = 0.59; 95% CI 0.36 to 0.98; number needed to treat (NNT) = 66; 95% CI 34 to 950; *P* = 0.04), compared with infants born to women allocated to Standard Care. This finding is consistent with our previous report of a significant 18% RR reduction in birth weight above 4 kg [24]. Furthermore, infants born to women allocated to Lifestyle Advice were shorter (birth length z-score −0,26 ± 0,76 versus −0,18 ± 0,80; adjusted difference in means −0,07; 95% CI −0,14 to −0,01; *P* = 0,04) than infants born to women allocated to Standard Care.

**Table 2:** **Infant outcomes by treatment group**

**Outcome**	**Lifestyle advice (n = 1075** ^**a**^ **)**	**Standard care (n = 1067** ^**a**^ **)**	**Unadjusted**		**Adjusted**	
			**Treatment effect (95% CI)**	***P*** **-value**	**Treatment effect (95% CI)**	***P*** **-value**
GA at birth, weeks^b^	39.29 ± 1.74	39.23 ± 2.07	0.06 (−0.10 to 0.23)	0.44	0.07 (−0.10 to 0.23)	0.42
Apgar score <7 at 5 minutes	22 (2.07)	22 (2.09)	0.99 (0.55 to 1.78)	0.98	0.99 (0.55 to 1.77)	0.97
Resuscitation required at birth	196 (18.23)	191 (17.89)	1.02 (0.85 to 1.22)	0.84	1.01 (0.85 to 1.21)	0.88
Birth weight, g^b^	3481 ± 554	3492 ± 613	−11.55 (−61.13 to 38.03)	0.65	−6.90 (−55.47 to 41.67)	0.78
Birth weight Z-score^b^	0.37 ± 1.03	0.43 ± 1.09	−0.06 (−0.15 to 0.03)	0.18	−0.05 (−0.14 to 0.03)	0.23
Birth length, cm^b^	49.84 ± 2.42	49.92 ± 2.84	−0.08 (−0.31 to 0.14)	0.48	−0.08 (−0.30 to 0.15)	0.51
Birth length Z-score^b^	−0.26 ± 0.76	−0.18 ± 0.80	−0.07 (−0.14 to −0.01)	0.03	−0.07 (−0.14 to −0.01)	0.04
Birth head circumference, cm^b^	34.77 ± 1.60	34.77 ± 1.90	0.00 (−0.15 to 0.15)	0.96	0.01 (−0.14 to 0.16)	0.92
Birth head circumference Z-score^b^	0.21 ± 1.03	0.26 ± 1.09	−0.05 (−0.14 to 0.04)	0.31	−0.05 (−0.14 to 0.04)	0.32
Birth weight ≥4.5 kg	23 (2.15)	39 (3.69)	0.58 (0.35, 0.97)	0 · 04	0 · 59 (0 · 36, 0 · 98)	0 · 04
Birth weight ≤2.5 kg	43 (4.03)	56 (5.29)	0.76 (0.51 to 1.13)	0.18	0.74 (0.50 to 1.09)	0.13
Ponderal index, kg/m^3b^	27.95 ± 2.85	27.82 ± 2.91	0.12 (−0.12 to 0.37)	0.33	0.12 (−0.12 to 0.36)	0.34
Predicted fat free mass, kg^b^	3.07 ± 0.38	3.08 ± 0.42	−0.01 (−0.04 to 0.02)	0.59	−0.01 (−0.04 to 0.03)	0.73
Admission to NICU ≥4 days	12 (1.12)	23 (2.18)	0.52 (0.26 to 1.03)	0.06	0.51 (0.26 to 1.02)	0.06
Admission to SCBU	388 (36.12)	382 (35.77)	1.01 (0.90 to 1.13)	0.87	1.00 (0.90 to 1.12)	1.00
Respiratory distress syndrome	13 (1.22)	27 (2.57)	0.47 (0.25 to 0.91)	0.03	0.47 (0.24 to 0.90)	0.02
Respiratory support	65 (6.09)	77 (7.20)	0.84 (0.61 to 1.16)	0.30	0.84 (0.61 to 1.15)	0.27
Moderate/severe respiratory disease	1 (0.09)	15 (1.42)	N/A	<0.001^d^	N/A	N/A
Discharged home on oxygen	1 (0.09)	3 (0.28)	N/A	0.37^d^	N/A	N/A
Patent ductus arteriosus	2 (0.19)	5 (0.47)	N/A	0.29^d^	N/A	N/A
Proven systemic infection	0 (0.00)	2 (0.19)	N/A	0.25^d^	N/A	N/A
Retinopathy of prematurity	1 (0.09)	4 (0.38)	N/A	0.22^d^	N/A	N/A
Necrotising enterocolitis	3 (0.28)	1 (0.09)	N/A	0.62^d^	N/A	N/A
Neonatal encephalopathy	0 (0.00)	0 (0.00)	N/A	N/A	N/A	N/A
Neonatal seizures	1 (0.09)	3 (0.28)	N/A	0.37^d^	N/A	N/A
Postnatal length of stay infant, days^e^	3.94 ± 7.26	4.41 ± 9.87	0.89 (0.82 to 0.97)	0.007	0.89 (0.82 to 0.97)	0.006

NICU, neonatal intensive care unit; SCBU, special care baby unit.

^a^Includes all live-born infants.

^b^Values are mean ± SD, and treatment effects are differences in means based on imputed data.

^c^Values are n(%), and treatment effects are relative risks based on imputed data.

^e^Values are mean ± SD, and treatment effects are ratios of means based on imputed data.

^d^ *P*-value derived Fisher’s exact test based on available data.

There was no statistically significant difference in infant admission to neonatal intensive care (Lifestyle Advice 1.12% versus Standard Care 2.18%; aRR = 0.51; 95% CI 0.26 to 1.02; *P* = 0.06). However, infants born to women following Lifestyle Advice were less likely to have respiratory distress syndrome (Lifestyle Advice 1.22% versus Standard Care 2.57%; aRR = 0.47; 95% CI 0.24 to 0.90; NNT = 75; 95% CI 40 to 532; *P* = 0.02), particularly moderate or severe respiratory disease (Lifestyle Advice 0.09% versus Standard Care 1.42%; *P* < 0.001), compared with infants born to women allocated to Standard Care (Table 2). Infants born to women in the Lifestyle Advice group also had a shorter postnatal length of hospital stay (3.94 ± 7.26 days versus 4.41 ± 9.87 days; adjusted difference in means 0.89; 95% CI 0.82 to 0.97; *P* = 0.006). There were no other statistically significant differences in infant outcomes identified between the groups.

Sensitivity analyses produced similar results, and did not alter the conclusions regarding the effectiveness of treatment in either the unadjusted or adjusted analysis for any outcome (data not shown).

## Discussion

Our findings indicate that provision of lifestyle advice to women who are overweight or obese during pregnancy is associated with a significant reduction in the risk of birth weight above 4.5 kg, in addition to a significant reduction in risk of respiratory distress syndrome, particularly moderate or severe disease, and a shorter postnatal hospital length of stay. Importantly, we did not identify any increase in the risk of harm, including low infant birth weight.

Our randomised trial has a number of strengths, including being the largest to date to evaluate the effect on clinically relevant neonatal outcomes of an antenatal lifestyle intervention for overweight or obese women. We utilised robust methodology, including blinding of outcome assessors and central randomisation, and achieved a high rate of infant follow-up and available birth outcome data.

Our trial is not without limitations. As highlighted previously [24], a potential limitation is the generalisability of our findings, with 60% of eligible women declining to participate (Figure 1). However, the demographic characteristics of women participating in the LIMIT trial are similar to the characteristics of the broader South Australian birthing population [32], providing reassurance that our findings are applicable in a wider clinical setting. It is also important to acknowledge that we report a number of secondary neonatal health outcomes. Although all were pre-specified, the study was not powered to identify differences in many of the secondary outcomes occurring relatively infrequently, and interpretation should therefore be with an element of caution.

The findings of a significant 41% RR reduction in birth weight above 4.5 kg among infants born to women following Lifestyle Advice compared with Standard Care is consistent with the 18% RR reduction in birth weight above 4.0 kg reported previously [24]. Immediate birth consequences associated with high infant birth weight are well recognised, and include shoulder dystocia and its sequelae, perinatal asphyxia, neonatal hypoglycaemia, need for nursery admission [33-36], and respiratory distress syndrome [37]. However, meta-analyses of population-based cohort studies indicate a longer-term association between high infant birth weight and an increased risk of both child [38,39] and adulthood overweight and obesity [40,41]. Observational data from 7,738 14-year-old adolescents in the United States Early Childhood Longitudinal Study [42] highlighted a significantly higher prevalence of obesity among children with birth weight above 4 kg. Whereas children of high birth weight represented 12% of the cohort, 36% of individuals who were obese at 14 years of age had birth weights over 4 kg [42]. Antenatal interventions that are successful in reducing the risk of high infant birth weight therefore represent a public health strategy of significant potential in tackling the increasing problem of overweight and obesity, both in the short and longer term [43,44]. The ongoing follow-up of infants born to women who participated in the LIMIT trial is therefore of great importance to evaluate the impact of reducing high infant birth weight on subsequent early childhood obesity.

We observed a 53% RR reduction in neonatal respiratory distress syndrome in infants born to women allocated the lifestyle intervention. This difference in neonatal respiratory distress syndrome was not explained by differences in the use of antenatal corticosteroids, or in differences in gestational age at birth. Some of this difference may reflect the observed 26% reduction in preterm birth and the 53% reduction in preterm pre-labour ruptured membranes (PPROM) among women in the intervention group [24], although these differences did not reach statistical significance. Although some authors have identified an increased risk of preterm birth in obese women [45], others indicate that this reflects iatrogenic prematurity rather than spontaneous labour [10]. In an analysis of the Danish National Birth Cohort, Nohr and colleagues identified an increased risk of preterm birth in obese women due to an increase in PPROM, which was postulated to reflect an increased risk of chorioamnionitis [46], although specific description of neonatal respiratory morbidity was not presented. Although we observed a significant reduction in risk of respiratory distress syndrome in infants born to women allocated to the lifestyle intervention, our findings do not suggest an aetiology related specifically to differences in risk of PPROM, chorioamnionitis or infectious causes [24].

Increasingly, there is recognition that although the consequences of preterm birth and prematurity can occur in a setting of clinical chorioamnionitis, effects are also evident following subclinical or histological inflammation [47]. However, the pathways affected and precise mechanisms remain to be determined, with evidence of an imbalance in the production of pro-inflammatory and anti-inflammatory cytokines [48]. There is increasing recognition that adipose tissue is far from an inert storage organ, being responsible for the active secretion of a number of metabolically active adipocytokines [49], and there is a well-described association in non-pregnant individuals between obesity and a low-grade inflammatory state [50,51], which, while speculative, may share similarities with subclinical chorioamnionitis.

## Conclusions

To our knowledge, our findings are the first to describe a significant reduction in neonatal respiratory morbidity among infants born to women who are overweight or obese following an antenatal dietary and lifestyle intervention. Furthermore, we postulate that this may be mediated by the significant improvements in maternal diet and physical activity following antenatal intervention, which we have reported previously [52]. It will be important to further consider specific dietary components and physical activity, and the impact these factors may have on maternal markers of inflammation, which are currently being evaluated through our prospectively established bio-bank.

Evidence to date about the effect of antenatal dietary and lifestyle interventions for women who are overweight or obese has focused on gestational weight gain, to the detriment of robust data describing both maternal and infant health outcomes [53]. Our randomised trial addresses this gap in the literature. Our findings indicate that providing an antenatal dietary and lifestyle intervention for women who are overweight or obese has health benefits for the infant, without increasing the risk of harm. Continued follow-up of participants, and ongoing interrogation of our bio-bank will be important to identify potential mechanistic pathways whereby changes to maternal diet and physical activity impact on clinical outcomes.
